# Clinical features of a Chinese female nongestational choriocarcinoma cohort: a retrospective study of 37 patients

**DOI:** 10.1186/s13023-020-01610-6

**Published:** 2020-11-18

**Authors:** Yuming Shao, Yang Xiang, Fang Jiang, Boju Pan, Xirun Wan, Junjun Yang, Fengzhi Feng, Tong Ren, Jun Zhao

**Affiliations:** 1 Department of Obstetrics and Gynecology, Peking Union Medical College Hospital, Chinese Academy of Medical Sciences and Peking Union Medical College, No. 1 Shuaifuyuan, Beijing, 100730 China; 2 Institute of Clinical Medicine, Peking Union Medical College Hospital, Chinese Academy of Medical Sciences and Peking Union Medical College, Beijing, China; 3 Department of Pathology, Peking Union Medical College Hospital, Chinese Academy of Medical Sciences and Peking Union Medical College, Beijing, China

**Keywords:** Trophoblastic disease, Choriocarcinoma, Nongestational choriocarcinoma, Germ cell tumor, Ovary

## Abstract

**Background:**

Choriocarcinoma is a rare malignant neoplasm, which is classified as either gestational choriocarcinoma or nongestational choriocarcinoma. The purpose of this study was to examine the clinical characteristics of Chinese female nongestational choriocarcinoma patients and discuss our experience in treating this rare disease.

**Results:**

We conducted a single-center retrospective study on a sample of 37 nongestational choriocarcinoma patients who were diagnosed and treated at Peking Union Medical College Hospital from March 1982 to March 2020. Their demographic, clinical, laboratory, and therapeutic data were collected. Detailed information was available for all 37 individuals in our sample. The primary lesions included 34 in the ovaries, 2 in the pituitary and 1 in the stomach. The median age of onset was 22 years, and the median follow-up period spanned 41 months. The lungs (40.5%) were the most commonly observed metastatic site. All subjects were treated with surgery and multidrug chemotherapies, and a median of 4.0 courses was required to achieve complete remission. The overall complete response rate, relapse rate, and 3-year and 5-year survival rates were 81.1%, 16.7%, 80.0%, and 75.5%, respectively.

**Conclusions:**

Nongestational choriocarcinoma can be managed well using surgery and multidrug chemotherapies, but the overall outcome of nongestational choriocarcinoma is still worse than that of gestational choriocarcinoma. Mixed nongestational choriocarcinoma seems to have similar therapeutic outcomes as pure tumors.

## Background

Choriocarcinoma is a rare and aggressive malignant tumor composed of biphasic cellular components with mononuclear cytotrophoblasts and multinucleated syncytiotrophoblasts [1]. The two significant choriocarcinoma subtypes, gestational choriocarcinoma (GC) and nongestational choriocarcinoma (NGC), have relatively different etiologies, biological activities and prognoses [2]. GC may arise following any type of pregnancy, including hydatidiform mole, term or preterm pregnancy, ectopic pregnancy, or abortion. GC is also chemosensitive, and the overall cure rate of these tumors is currently > 90% [3]. In contrast, NGC may develop either from germ cells in the gonads or extragonadal locations, or it can rarely occur in parenchymal organs due to dedifferentiation of somatic carcinoma [1]. The former subset of tumor is classified as a type of germ cell tumor.

Owing to the rarity of NGC, its clinical characteristics remain unclear, and the symptoms are often nonspecific. Abnormal uterine bleeding, abdominal pain, endocrine disorders, detection of a pelvic mass and precocious puberty have been previously reported [4, 5]. Germ cell tumor NGCs occur in the ovaries and midline locations, such as the mediastinum, retroperitoneum, and pineal gland. Uterus, cervix, stomach, pancreas and rectum have also been reported as locations of NGCs with or without somatic carcinoma components in several case reports [6–10]. Some studies have concluded that NGC is less sensitive to chemotherapy and has a relatively poor prognosis compared to GC [11]. In those case reports, therapeutic responses varied. Further large NGC cohorts are still needed to verify these conclusions.

To the best of our knowledge, there has been no report of a NGC cohort from another medical center except for our previously reported clinical analysis of 21 nongestational ovarian choriocarcinomas in 2009 [4]. Our previous study on cases from March 1982 to October 2008 indicated that the rates of complete remission and 5-year overall survival were 76.2% and 79.4%, respectively. Due to gradually increased understanding of this disease, several new NGC cases have been diagnosed in the past 11 years at our medical center.

In this study, we reviewed the medical records of all NGC patients treated at our institution. The purpose of this study was to demonstrate the clinical characteristics of Chinese female nongestational choriocarcinoma patients and discuss our experience in treating this rare disease.

## Methods

Since GC and NGC have similar histological features, a diagnosis of NGC should combine histopathological results with patient clinical characteristics, so this study adopted modified Saito’s NGC diagnostic procedures. Saito’s NGC diagnostic criteria take both aspects into consideration and include four items as follows, (1) absence of disease in the uterine cavity, (2) pathological confirmation of choriocarcinoma with persistent elevated serum levels of β-human chorionic gonadotropin (β-hCG), (3) exclusion of molar pregnancy, and (4) exclusion of coexisting intrauterine pregnancy [12]. In addition to Saito’s criteria, two more exclusion criteria were included as follows: (1) history of previous ectopic pregnancies and (2) lesions were only observed in lung with no lesions in other organs.

We retrospectively reviewed the medical records of 37 NGC patients recruited between March 1982 and March 2020 at Peking Union Medical College Hospital (PUMCH). Written informed consent was obtained from each patient, and this study was approved by the Institutional Review Board of PUMCH.

Detailed demographic and clinical data were collected. Clinical data included initial symptoms, sexual contact history, comorbidities, and family history of tumor. Laboratory values of β-hCG and alpha-fetoprotein (AFP) were reviewed as available. Therapeutic modalities included surgery, chemotherapy and radiotherapy. The number of chemotherapy courses was carefully calculated.

Response of each patient to therapeutic modalities was categorized into the following subsets according to the World Health Organization 1981 Standardization of reporting results of cancer treatment and our former study [4, 13]: complete response (CR), partial response (PR), progressive disease (PD) and relapse. CR was defined as complete elimination of all clinical syndromes and no laboratory or imaging abnormalities for at least 4 weeks. PR was noted as a decrease in β-hCG titer to less than 50% of its original level and an estimated decrease in tumor size of 50% or more for at least 4 weeks. PD is defined as a rising hCG level of at least 25% with development of previous lesions or occurrence of new metastases during treatment. Relapse was listed for patients with elevated β-hCG who had reached CR with a treatment-free interval of at least 3 months.

All data were recorded and analyzed in SPSS statistics software version 22. Descriptive data are expressed as numbers (%) for categorical variables and as medians (range) for continuous variables. Student's t-test was used to analyze both continuous and categorical variables. All tests were two-sided, and a *p* value of less than 0.05 was considered statistically significant. Kaplan–Meier methods were applied for survival analysis.

## Results

### Demographic data

A total of 37 NGC patients were recruited, all of Chinese origin. The median age at disease onset was 22 (range 9–44) years. As of March 2020, the follow-up period of this cohort amounted to 41 (range 1–269) months. Twenty-three patients had no pregnancy history, including 7 premenarchal girls, and 20 patients declared no previous history of sexual activity (Table 1).

**Table 1:** Demographic and clinical characteristics of the 37 patients with nongestational choriocarcinoma

Demographic characteristics	N = 37
Age at diagnosis, median (range), years	22 (9–44)
< 15 years old	9 (24.3%)
16–20 years old	9 (24.3%)
21–25 years old	5 (13.5%)
25–30 years old	5 (13.5%)
> 30 years old	9 (24.3%)
Duration of follow-up, median (range), months	41 (1–269)
Primary location	
Ovary	34 (91.9%)
Left	11 (32.3%)
Right	22 (64.7%)
Bilateral	1 (2.9%)
Pituitary	2 (5.4%)
Stomach	1 (2.7%)
Metastatic lesion	
Lung	15 (40.5%)
Brain	2 (5.4%)
Liver	2 (5.4%)
Extensive pelvic metastases	4 (10.8%)
Extensive abdominal metastases	4 (10.8%)
Initial symptoms	
Abdominal pain	24 (64.8%)
Acute abdomen	6 (25.0%)
Abnormal uterine bleeding	16 (43.2%)
Insipidus	2 (5.4%)
Pregnancy symptoms	3 (8.1%)
Palpable abdominal mass	2 (5.4%)
Hemoptysis	1 (2.7%)
Cough	1 (2.7%)
Fever	1 (2.7%)
Headache	1 (2.7%)
Melena	1 (2.7%)
Laboratory tests	
Serumβ-hcg, median (range), mIU/ml	15,126.7 (89.1–386,274)
AFP, elevated	1/13 (7.7%)
Staging of ovarian NGC patients	
Ovarian cancer staging^a^	N = 34
I	14 (41.2%)
II	2 (5.9%)
III	2 (5.9%)
IV	16 (47.1%)
Choriocarcinoma staging^b^	N = 34
II	14 (41.2%)
III	11 (32.4%)
IV	9 (26.5%)

*β-hCG* β-human chorionic gonadotropin, *AFP* alpha-fetoprotein, *NGC* nongestational choriocarcinoma

^a^According to FIGO 2013 staging standard of ovarian cancer

^b^According to FIGO 2000 staging standard of choriocarcinoma

### Histological data

All 37 patients underwent surgery and thus had a histopathological confirmation of choriocarcinoma. Thirty patients exhibited pure type NGC with no other germ cell tumor components. A mixture of mononucleated cells and variable levels of multinucleated syncytiotrophoblasts were observed. Tumor cells typically surrounded a central area of necrosis and hemorrhage. Three cases had primarily necrosis with limited tumor cells on the periphery. Immunohistochemistry results for hCG, HSD3B1, CD10, CD146 and HLA-G were usually positive. Seven patients presented with histopathologically confirmed mixed type NGC (1 in pituitary, 5 in ovary, 1 in stomach). Apart from choriocarcinoma, other components included dysgerminoma, embryonal carcinoma, teratoma and adenocarcinoma.

### Clinical presentations

For 37 NGC patients, ovary was the most commonly affected location, with ratio of 11:22:1 for left:right:bilateral. Two patients had NGC in the pituitary, and another presented with NGC in the stomach. Twenty-one (56.8%) patients had metastatic lesions of which lungs were the most commonly observed (40.5%) location. Other locations of metastatic lesions included the brain in two patients, and the liver in two patients. Two patients had extensive lesions in both the abdomen and pelvic cavity. Another two patients only had extensive metastasis in the abdominal cavity, and two only had extensive pelvic cavity metastases.

Symptoms of these NGC patients were relatively nonspecific. Abdominal pain was reported by 24 patients, 6 of whom presented with acute abdominal pain and underwent emergency surgery. Sixteen postpubertal patients presented with abnormal uterine bleeding, such as irregular menstruation and amenorrhea. Two pituitary patients presented with insipidus. Other tumor-related manifestations, such as fever, pregnancy symptoms, palpable mass, headache, cough, hemoptysis, and melena were only rarely observed. No choriocarcinoma or any other type of tumor history in primary relatives were reported by these 37 NGC patients.

Serum β-hCG levels of each patient were regularly measured. The median of each patient’s highest value of serum β-hCG during the whole disease course was 77,278 (range 89.1–386,274) mIU/ml. AFP was tested in 13 patients. Only one mixed germ cell NGC tumor with dysgerminoma and embryonal carcinoma exhibited elevated AFP.

Staging of NGC remains unclear. There are no standard guidelines for gastric and pituitary NGC staging. For 34 ovarian NGC subjects in this study, 14 were in stage I, 2 were in stage II, 2 were in stage III, and 16 were in stage IV, according to the International Federation of Gynecology and Obstetrics (FIGO) 2013 standard of ovarian cancer, with a 5-year survival rate of 92.9%, 100%, 0% and 68.8%, respectively. In view of FIGO’s 2000 classification of choriocarcinoma, 14 were in stage II, 11 in stage III, and 9 in stage IV. The CR rates for these cases were 92.9%, 81.8%, and 44.4%, respectively (Table 1).

### Therapeutic modalities

Treatments included chemotherapy, surgery, radiotherapy and intrathecal injection. All patients received multiple-drug combined chemotherapy. The primary regimens chosen were EMA/CO (Etoposide, Methotrexate, Actinomycin D, Cyclophosphamide, Vincristine for 20 patients), FAEV (Floxuridine, Actinomycin-D, Etoposide, Vincristine for 15 patients), BEP (Bleomycin, Etoposide, Cisplatin for 7 patients), PVB (Bleomycin, Vincristine, Cisplatin for 4 patients), and ICE (Ifosfamide, Carboplatin, Etoposide for 2 patients). Nine patients received chemotherapy prior to surgery. The overall median courses and courses to reach CR were 7.5 and 4.0, respectively. Three to four consolidation chemotherapies were recommended after hCG levels had returned to normal. For 7 mixed NGC patients, 2 primarily received the BEP protocol and another 3 were administered the EMACO protocol, all of whom exhibited CR. BEP, EMACO and FAEV protocols were prescribed for another ovarian mixed NGC patient but were unable to control her disease. The stomach mixed NGC patient obtained CR after FAEV therapy but experienced disease relapse 2 years later.

Twelve patients showed drug resistance to primary chemotherapy. Five patients were resistant to FAEV chemotherapy. EMACO, PEB, and ICE regimens were then prescribed for these different patients. Two patients exhibited resistance to the EMACO regimen but responded to the FAEV regimen. Two patients were prescribed the PEB/PVB regimen prior to being seen in our clinic, at which time they received the EMACO/FAEV regimen due to unsatisfactory decreases in hCG levels. Another 3 patients were resistant to nonstandard multiagent chemotherapies.

Myelosuppression was a commonly observed adverse effect of chemotherapy, but only one patient experienced life-threatening myelosuppression. Other conditions, such as liver injury, dental ulcer, and anorexia, were rarely reported by sporadic patients.

All 37 patients underwent surgery. Of 34 ovarian NGC patients, 16 underwent cytoreductive surgery, and 18 received fertility-preserving surgery. The scale of cytoreductive surgery includes the uterus, bilateral ovary, bilateral fallopian tube, omentum, pelvic and para-aortic lymph nodes, appendix, and any other abdominal/pelvic metastases. Fertility-preserving surgery preserved the uterus, unilateral ovary, and unilateral fallopian tube, including unilateral salpingo-oophorectomy, unilateral salpingo-oophorectomy with partial omentectomy, and unilateral salphingo-oophorectomy with pelvic and para-aortic lymphadenectomy, omentectomy, and appendectomy. Among 16 patients who received cytoreductive surgery, 6 initially underwent fertility-preserving procedures. However, a debulking surgery was performed at our medical center because of unsatisfactory decreases in β-hCG or due to disease relapse. Even though 15 patients had lung metastases, only three of them received pulmonary lobectomy. The gastric NGC patient received subtotal gastrectomy with lymphadenectomy.

As an adjunct to chemotherapy, one pituitary NGC patient completed 25 radiotherapy treatments (total 45 Gy). Another patient with cerebral metastasis received 3 methotrexate intrathecal injections (Table 2).

**Table 2:** Treatment and outcome of the 37 patients with nongestational choriocarcinoma

Surgical operations	
Laparotomy	31 (83.8%)
Laparoscopic surgery	4 (10.8%)
Craniotomy	2 (5.4%)
Chemotherapy	
Total number of courses, median (range)	7.5 (1–43)
Number of courses before CR, median (range)	4.0 (1–27)
Regimen	
EMACO	20 (54.1%)
FAEV	17 (45.9%)
BEP	7 (18.9%)
PVB	4 (10.8%)
ICE	2 (5.4%)
Outcome	
CR	30 (81.1%)
Relapse	5 (16.7%)
PR	4 (10.8%)
PD	3 (8.1%)

*CR* complete response, *PR* partial response, *PD* progressive disease, *EMACO* Etoposide, Methotrexate, Actinomycin D, Cyclophosphamide, and Vincristine, *FAEV* Floxuridine, Actinomycin-D, Etoposide, and Vincristine, *BEP* Bleomycin, Etoposide, and Cisplatin, *PVB* Bleomycin, Vincristine, and Cisplatin, *ICE* Ifosfamide, Carboplatin, and Etoposide

### Outcomes

Among all 37 NGC patients, 30 (81.1%) achieved CR, and 4 (10.8%) achieved PR. Three (8.1%) patients experienced PD and died. One patient was diagnosed in the 1980s and received nonstandard multidrug combined chemotherapy. She gave up treatment after 44 months of unsuccessful decreases in hCG. Another subject had mixed NGC with teratoma and dysgerminoma components. She underwent debulking surgery and received FAEV, BEP and EMACO chemotherapy for 23 months. The last patient died of chemoresistance. Successive regimens of FAEV, PVB, ICE and EMAEP (Etoposide, Methotrexate, Actinomycin D, Cisplatin) were unable to decrease β-hCG levels to normal.

We require regular follow-up in our clinic, including clinical evaluation (symptoms and pelvic examination), assessment of β-hCG levels and imaging. The schedule was as follows: once monthly for 3 months, every 3 months for 9 months, every 6 months for 2 years, once a year for 2 years, and every 2 years thereafter.

For 34 CR and PR patients, 8 patients were lost to follow-up, including 4 CR and 4 PR patients. With a median follow-up of 41 months, overall 1-year, 3-year and 5-year survival rates were 86.2%, 80.0% and 75.5% respectively.

Five (16.7%) CR patients experienced disease relapse during follow-up after achieving CR for 4, 6, 22, 24 and 31 months. Four were affected by ovarian NGC and initially received fertility-preserving procedures. After disease relapse, three underwent another debulking surgery and subsequent chemotherapies. One patient only received another 6 courses of EMACO. All four attained CR again. The stomach NGC patient showed multiple metastases in the abdomen 2 years after reaching CR and subsequently died of chemoresistance.

### Comparison of pure and mixed type NGCs

**Fig. 1:**
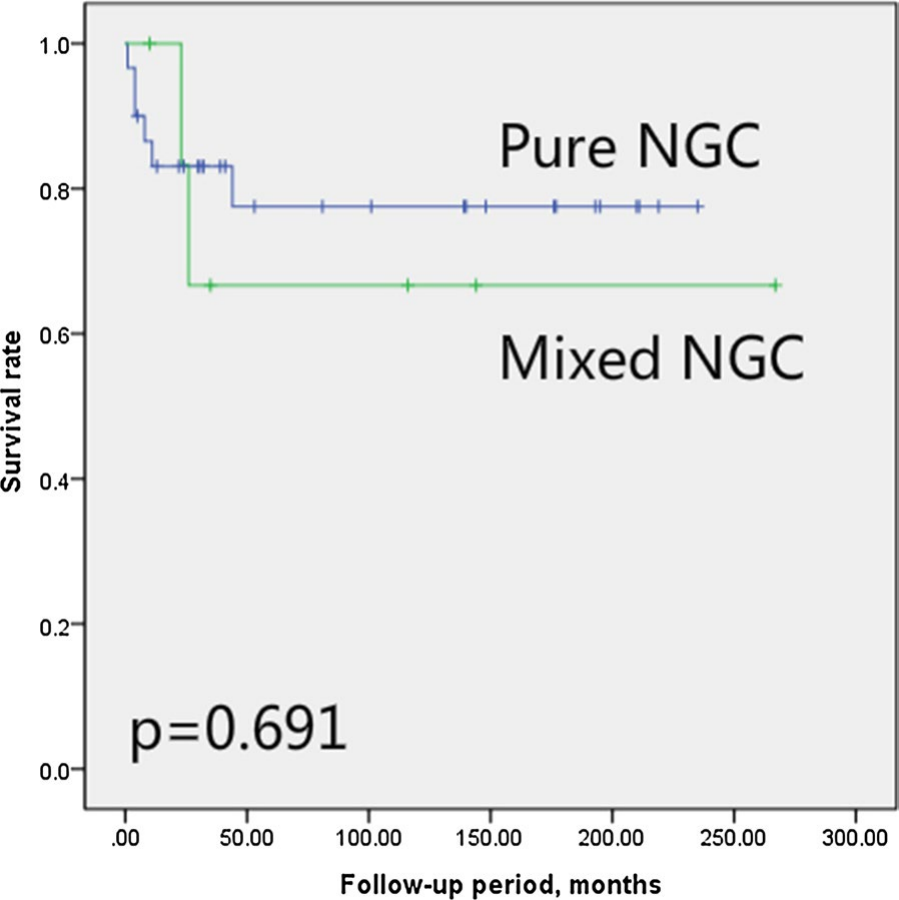
Survival rates of mixed nongestational choriocarcinoma patients and pure ones. *NGC* nongestational choriocarcinoma

Student's t-test revealed that mixed and pure NGCs demonstrated no significant differences with respect to age of onset (*p* = 0.283), choriocarcinoma staging (*p* = 0.245), ovarian cancer staging (*p* = 0.507, only for 34 ovarian NGC patients), β-hCG level (*p* = 0.311), presence/absence of metastasis (*p* = 0.523), overall courses of chemotherapy (*p* = 0.836), courses to reach CR (*p* = 0.262), or CR rate (*p* = 0.277) (Fig. 1).

## Discussion

To date, this is the largest reported NGC retrospective cohort. The median follow-up period of this study was 41 (range 1–269) months. The clinical and demographic data as well as treatment plans of 37 NGC patients were reviewed in detail. According to the data from this study, the ovary and lung were the most commonly observed primary and metastatic sites, respectively. Mixed NGCs seemed to have similar therapeutic outcomes compared to pure NGCs. When managed with surgeries and multidrug chemotherapies, the overall 3- and 5-year survival rates for NGC were 80.0% and 75.5%, respectively.

NGC is seldom reported due to its low incidence. Available publications are primarily case reports and occasionally a case series of patients. Lung metastases were commonly observed in NGC patients. Liver, brain, pelvic and abdominal metastases were present only in limited cases. This metastasis pattern is consistent with this study and our former research [4]. Some studies have shown that mixed type NGCs seem to have relatively poor prognosis [1]. However, this conclusion was not verified in an NGC cohort. Our study showed that mixed type and pure type NGCs exhibited similar CR and survival rates. Moreover, the overall prognosis of NGC was good, and the 5-year survival rate was still high, similar to our former study of 79.4% [4].

It is difficult to discriminate whether a subject has GC or NGC due to the similar macroscopic appearance, histopathological features, and elevated serum levels of β-hCG seen in these two diseases [1]. Notably, higher serum β-hCG levels do not ensure choriocarcinoma components of a mixed germ cell tumor [14, 15]. Since NGC and GC cannot be histologically distinguished, this study primarily used Saito’s 1960s NGC clinical criteria [12]. Moreover, two more exclusion criteria were added to exclude some possible GC patients. First, due to hematogenous spread of GC, primary lesions in the uterine cavity may spontaneously regress, but metastasis to the lungs may still occur [16–18]. On the other hand, since lung is not a common site of germ cell tumors, patients with solitary lesions in the lungs but no lesions in other organs were most likely to be GC metastasis patients and were excluded accordingly. Second, there is a small possibility that some ovarian NGCs may originate from ectopic ovarian pregnancies. Even though ovarian pregnancy is one of the rarest forms of ectopic pregnancy, having an incidence of 1/7000–1/40,000 in live births and only 0.5–3% of all ectopic gestations, patients with previous ectopic ovarian pregnancy were also excluded [19].

Some studies insist that NGC patients should have no pregnancy history [1]. A choriocarcinoma must be NGC if technically excluding a history of pregnancies and even previous sexual activity. However, most NGCs belong to germ cell tumors. The pathogenesis of germ cell tumors is not related to pregnancy [20]. Therefore, patients may have a previous pregnancy that is unrelated to NGC. The diagnostic criterion of no pregnancy is strict indeed, but not comprehensive enough and may omit some gonadal or extragonadal germ cell tumor NGCs. In this study, only 14 NGC patients reported previous sexual activity.

DNA polymorphism analyses are also an important tool for diagnosis. Genotyping can identify the presence of a parental allele, confirming a diagnosis of GC, since NGC has only maternal alleles. Based on this distinguishing feature, genetic analysis is applied by increasing studies and is not considered controversial [21, 22]. However, since genetic analysis is not widely used for choriocarcinoma patients, clinical diagnosis should still be the primary method.

Because of its rarity, the staging of NGC remains unclear. Since ovarian NGC is a specific type of ovarian germ cell malignancies, most cases applied ovarian cancer staging for ovarian NGC. In this study, eleven of the ovarian NGC patients experienced lung metastases, and were classified as stage IV based on ovarian cancer criteria. Nine of them achieved CR, and only one patient died because of irregular chemotherapy. Another one was lost to follow up after 4 months of treatment. Thus, the overall prognosis for patients with ovarian NGC and lung metastasis were relatively good. However, the reported survival rate of stage IV ovarian germ cell malignancies is only 14–54% [23]. This inconsistency shows a possible weakness for ovarian cancer staging for ovarian NGC. On the other hand, some features of NGC are also commonly observed in GC, such as a high rate of lung metastases (40.5% in this cohort), response to therapy, and optimistic prognosis. Therefore, this study also adopted and recommended choriocarcinoma staging classification. Under this classification, the prognosis of NGC was slightly worse than that of GC (CR rates: 99.3%, 89.4%, and 79.0% for stage II, III, and IV GC, respectively) [24]. However, the WHO prognostic scoring system for malignant gestational trophoblastic diseases was not suitable for NGC. Two items of the prognostic scoring system, antecedent pregnancy and interval from index pregnancy, were inappropriate criteria for evaluation.

Currently there are no standard therapies for NGC, and instead, treatments primarily follow that of GC [25]. NGC is thought to originate from the patient herself, so surgical procedures, along with chemotherapies, are required in most cases [26]. EMA/CO and FAEV regimens were the most often chosen multiple-drug combined chemotherapies in this cohort, showing good tolerance and efficacy for NGC. Neoadjuvant chemotherapies should also be considered for patients with high β-hCG levels. Both BEP and EMA/CO regimens seem to be effective in controlling mixed germ cell tumors with choriocarcinoma components, and overall CR and survival rates were not different between mixed choriocarcinoma patients and pure tumors. Since NGC primarily affects young premenopausal women who have not completed childbearing, every effort is made to minimize the long-term effects of cancer treatment. Fertility-preserving surgeries for patients with localized lesions could also be considered.

This study has some limitations. First, it was a retrospective study, which may implicate recall and missing data bias, so we utilized different methods to gather information. Second, the number of patients in our cohort was still relatively small, even though NGC is such a rare disease globally. Last, as a top national medical center in China, patients from all over China come to our center, and we might be presented with more severe cases that might not be clinically representative.

## Conclusions

In summary, the samples in this study comprised a Chinese nongestational choriocarcinoma cohort. We provided detailed demographic and clinical data on these patients. Our study demonstrated that mixed NGC seems to have similar therapeutic outcomes compared to pure NGC. NGC can be managed well with surgery and multidrug chemotherapies, but the overall treatment response is still worse than the response of GC. Our cohort study adds to the knowledge of the disease spectrum of trophoblastic diseases and will hopefully aid researchers in deepening their understanding of this illness. Further studies with more enrolled cases are needed to verify our results.

## Data Availability

The datasets used and analyzed during the current study are available from the corresponding author on reasonable request.

## Abbreviations

GC: Gestational choriocarcinoma
NGC: Nongestational choriocarcinoma
CR: Complete response
β-hCG: β-Human chorionic gonadotropin
PUMCH: Peking Union Medical College Hospital
AFP: Alpha-fetoprotein
PR: Partial response
PD: Progressive disease
EMACO: Etoposide, Methotrexate, Actinomycin D, Cyclophosphamide, Vincristine
FAEV: Floxuridine, Actinomycin-D, Etoposide, Vincristine
BEP: Bleomycin, Etoposide, Cisplatin
PVB: Bleomycin, Vincristine, Cisplatin
ICE: Ifosfamide, Carboplatin, Etoposide
EMAEP: Etoposide, Methotrexate, Actinomycin-D, Cisplatin
FIGO: International Federation of Gynecology and Obstetrics
